# Soluble intercellular adhesion molecule-1 is associated with hepatocellular carcinoma risk: multiplex analysis of serum markers

**DOI:** 10.1038/s41598-017-10498-5

**Published:** 2017-09-11

**Authors:** Vincent L. Chen, An K. Le, Ondrej Podlaha, Jacqueline Estevez, Biao Li, Philip Vutien, Ellen T. Chang, Yael Rosenberg-Hasson, Stefan Pflanz, Zhaoshi Jiang, Dongliang Ge, Anuj Gaggar, Mindie H. Nguyen

**Affiliations:** 1 Department of Medicine, Stanford University Medical Center, Palo Alto, CA USA; 2 Division of Gastroenterology, University of Michigan Health System, Ann Arbor, MI USA; 3 Division of Gastroenterology and Hepatology, Stanford University Medical Center, Palo Alto, CA USA; 4 Gilead Sciences, Foster City, CA USA; 5 Boston University School of Medicine, Boston, MA USA; 6 Department of Medicine, Rush University Medical Center, Chicago, IL USA; 7 Department of Health Research and Policy (Epidemiology), Stanford University School of Medicine, Stanford, CA USA; 8 Institute for Immunity, Transplantation, and Infection Operations, Stanford University School of Medicine, Stanford, CA USA

## Abstract

Individualized assessment of hepatocellular carcinoma (HCC) risk in chronic liver disease remains challenging. Serum biomarkers including cytokines may offer helpful adjuncts to standard parameters for risk prediction. Our aim was to identify markers associated with increased HCC incidence. This was a prospective cohort study of 282 patients with both viral or non-viral chronic liver disease. Baseline serum cytokines and other markers were measured in multiplex with a commercially-available Luminex-based system. Patients were followed until death or HCC diagnosis. We performed Lasso-based survival analysis to determine parameters associated with HCC development. Cytokine mean florescence intensity (MFI) was the primary predictor and HCC development the primary outcome. 25 patients developed HCC with total follow-up of 1,363 person-years. Parameters associated with increased HCC incidence were cirrhosis, hepatic decompensation, and soluble serum intercellular adhesion molecule 1 (sICAM-1) MFI. No other molecules increased predictive power for HCC incidence. On univariate analysis, the parameters associated with HCC incidence in patients with cirrhosis were age, antiviral treatment, and high sICAM-1 MFI; on multivariate analysis, sICAM-1 remained associated with HCC development (adjusted HR = 2.75). On unbiased screening of serum cytokines and other markers in a diverse cohort, baseline sICAM-1 MFI is associated with HCC incidence.

## Introduction

Liver cancer, of which hepatocellular carcinoma (HCC) constitutes 70–85%, is the fifth most common cancer and second most common cause of cancer death worldwide, and was responsible for approximately 700,000 deaths in 2008^1, 2^. HCC incidence is rising in the United States and worldwide^1, 3^. While HCC is known to arise in the setting of chronic liver disease, such as infection with hepatitis B virus (HBV) or hepatitis C virus (HCV)^4, 5^, and there have been major advances in our understanding of HCC tumorigenesis in recent years, HCC pathogenesis remains incompletely and poorly understood. This is particularly true in regards to potential differences in pathogenesis based on etiology of underlying liver disease, which may have contributed to the lack of a universal risk prediction model for HCC based on currently available clinical parameters. Minimally invasive serum biomarkers to assess HCC risk have potential utility as adjuncts to standard clinical risk assessment^6, 7^.

Prior studies suggest that several cytokines have important effects in the pathogenesis of and survival after HCC. Interleukin (IL)-6 is one of the best studied in this context. In murine HCC models, IL-6 produced in either an autocrine or paracrine fashion by hepatic macrophages drives HCC tumorigenesis^8–10^. Interestingly, IL-6 inhibition also eliminated the increased risk of HCC in male mice^10^. Clinically, serum IL-6 concentration in HCC patients correlates with larger tumor size and decreased survival^11, 12^; and among at-risk patients with chronic hepatitis B and C^13–16^. Elevated serum IL-10 is also associated with poorer survival in HCC patients^17, 18^, possibly related to inhibition of γδ-T cell and dendritic cell function^19, 20^. More recently, soluble proteins associated with angiogenesis have been found to correlate with survival after HCC diagnosis^21^.

Intercellular adhesion molecule (ICAM-1) is an adhesion molecule that induces leukocyte trafficking to endothelial cells^22, 23^. ICAM-1 is also expressed on HCC cancer stem cells^24^. The soluble form of ICAM-1, sICAM-1, is a counter-receptor for the integrin lymphocyte function-associated antigen 1 and inhibits leukocyte trafficking^22^. sICAM-1 inhibits lymphocyte adhesion to major histocompatibility complex-restricted tumor-specific T cell immunity and promotes angiogenesis^25, 26^. In humans, high sICAM-1 concentration is associated with larger HCC tumor size^27^ and increased risk of HCC recurrence following resection^28^. High sICAM-1 concentration may also correlate with HCC incidence, based on one small study of 99 HCV patients^29^. It is also associated with poor prognosis in other malignancies such as breast cancer^30^ and non-Hodgkin lymphomas^31^.

These findings indicate that specific cytokines and other serum peptides are important in HCC tumorgenesis and are also associated with disease severity, and thus may be attractive targets in biomarker development. However, data on the use of cytokines to assess risk of HCC and other consequences of chronic liver disease remain highly limited. We hypothesized that serum cytokines correlate with HCC incidence and mortality in chronic liver disease, independently of known clinical risk factors or underlying etiology of liver disease. In order to investigate this question, we performed a prospective cohort study of HCC-free patients to determine how baseline cytokine profile may influence future risk of HCC.

## Patients and Methods

### Study design and patient population

We performed a prospective cohort study of 282 patients with chronic liver disease who were enrolled at liver clinics at Stanford University Medical Center between 2004 and 2010. This was a convenience cohort. Only patients with HBV, HCV, alcohol, nonalcoholic fatty liver disease, or cryptogenic liver disease were included; patients with HBV-HCV or HBV-HDV or HIV coinfection were not included, since as part of another study we were interested in characterizing immune phenotypes in individual causes of liver disease. Patients with a prior history of HCC and those diagnosed with HCC within 12 months of enrollment were excluded. Patients were followed approximately every 6 months until death or HCC development or end of study follow-up. HCC diagnosis was determined by CT or MRI imaging based on the 2011 American Association for the Study of Liver Disease guidelines^5^. Cirrhosis was defined as having pathological evidence of fibrosis stage 4, clinical evidence of portal hypertension (platelet count <120,000/μL, otherwise-unexplained splenomegaly, ascites, or gastroesophageal varices on imaging), prior hepatic decompensation (hepatic encephalopathy, ascites, variceal gastrointestinal bleeding), or laboratory evidence of decreased synthetic function (total bilirubin >2.0 mg/dL or INR > 1.2 without alternative explanation). Hepatic decompensation included patients with cirrhosis and one of the following decompensating events: variceal bleeding, hepatic encephalopathy, or ascites. Serum specimens were collected and questionnaires were administered by research coordinators at the time of enrollment. Additional patient characteristics were also collected by individual chart review. All patients provided written informed consent. This study was approved by the Institutional Review Board at Stanford University (Stanford, CA). All experiments were performed in accordance with relevant guidelines and regulations.

### Cytokine multiplex analysis

Serum specimens were submitted to the Stanford Human Immune Monitoring Core for analysis of cytokines and other serum biomarkers using an FDA-approved Luminex 200-based platform (Austin, TX). In brief, human 51-plex plates were purchased from Affymetrix and used according to the manufacturer’s recommendations with modifications as described below. Samples were mixed with antibody-linked polystyrene beads on 96-well filter-bottom plates and incubated at room temperature for 2 h followed by overnight incubation at 4 °C. Room temperature incubation steps were performed on an orbital shaker at 500–600 rpm. Plates were vacuum filtered and washed twice with wash buffer, then incubated with biotinylated detection antibody for 2 h at room temperature. Samples were then filtered and washed twice as above and re-suspended in streptavidin-phycoerythrin. After incubation for 40 minutes at room temperature, two additional vacuum washes were performed, and the samples re-suspended in Reading Buffer. Each sample was measured in duplicate. Plates were read using a Luminex 200 instrument with a lower bound of 100 beads per sample per cytokine. Custom assay Control beads by Radix Biosolutions were added to all wells. This multiplex system is based on a flow cytometer and allows detection of up to 100 cytokines in a single 96-well plate.

A total of 51 serum cytokines and other biomarkers were measured simultaneously. The cytokines were: cluster of differentiation (CD) 40 ligand, epithelial cell-derived neutrophil-activating peptide-78, eotaxin, fibroblast growth factor-basic, granulocyte colony stimulating factor, granulocyte macrophage colony stimulating factor, growth-regulated oncogene-α, hepatocyte growth factor, sICAM-1, interferon-α, -β, and -γ, interleukin (IL)-10, IL-12 p40, IL-12 p70, IL-13, IL-15, IL-17A, IL-17F, IL-1α, IL-1β, IL-1RA, IL-2, IL-4, IL-5, IL-6, IL-7, IL-8, interferon-γ-induced protein-10, leptin, leukemia inhibitory factor, macrophage, monocyte chemotactic protein-1 and-3, monocyte induced by gamma interferon, macrophage inflammatory protein-1α and -1β, nerve growth factor, plasminogen activator inhibitor-1, platelet derived growth factor-BB, chemokine (C-C motif) ligand 5, resistin, stem cell factor, soluble Fas ligand, transforming growth factor-α and -β, tumor necrosis factor-α and-β, tumor necrosis factor-related apoptosis-inducing ligand, soluble vascular cell adhesion molecule-1, and vascular endothelial growth factor. Several of these proteins overlap with previously-published cancer hallmark-based gene signatures, including IL-2, IL-8, and tumor necrosis factor-α^32^. Each plate contained two replicates of a “control” serum (taken from a middle-aged Caucasian male), calibration samples to aid in converting median fluorescent intensity values to units of concentration, and CHEX quality control beads designed to assist in detecting experimental failures. Specific anti-cytokine antibodies linked to unique polystyrene beads were applied to each 25-μl sample. Each plate was read on a Luminex reader, which identifies and classifies individual analytes by their bead color using the red laser, and quantifies analyte levels using the excitation of the green laser. Data were imported into Masterplex software and analyzed to obtain 51 standard curves for each analyte. Data are presented as median fluorescence intensity (MFI).

The rationale for presenting MFI rather than concentration is as follows. Detection for sICAM1 and other biomarkers in the samples are close to the upper limit on the standard curves. Since this panel measures 51 cytokines in a multiplex setup, the dilution is not optimal for all biomarkers. Therefore, it is preferable to use MFI values to prevent any extrapolation and add distortion with use of pg/ml values.

### Statistical Analysis

#### Analysis of clinical characteristics

Descriptive statistics were reported as proportion (%) for categorical variables, and mean ± standard deviation (SD) or median (and interquartile range) for continuous variables. Analyses of normally distributed continuous variables were performed using the Student *t* test. Nonparametric statistics were applied when continuous variables were not normally distributed. The chi-square test was used to evaluate categorical variables. Lasso-based survival analysis was used to determine the parameters most strongly correlated with HCC development. The primary predictor variables were serum MFI of the individual cytokines and the primary outcome was development of HCC. Subsequent analysis with these parameters was performed using Kaplan-Meier methods. The log-rank test was used to compare survival between independent subgroups. The Cox proportional hazard model was used to estimate the hazard ratio (HR) relating risk factors to HCC incidence. Statistical significance was defined as a 2-tailed *P* value < 0.05. Survival analysis and time-dependent receiver operating characteristics (ROC) analysis was performed in R. All other statistical analyses were performed using Stata 11.0 (Stata Corporation, College Station, TX).

#### Analysis of HCC incidence based on cytokine profiles

To identify the most important clinical and molecular factors that influence the incidence of HCC, we utilized a Lasso-based survival method (R package glmnet). This method generates a collection of Cox proportional hazard models with increasing number of covariables and generally better performance. A key feature of Lasso-based survival analysis is that in each iteration the complexity of parameter space is contained, and then the optimal set of covariables are determined under such constraint through efficient optimization algorithms. By varying the complexity of parameter space from small to large, we increase the number of covariables in each model gradually. Therefore, covariables that enter models in early steps are the most prominent when only a few covariables are allowed in the modeling as they fit better models by explaining a larger amount of variance in the data than those unselected or selected in later steps.

This model included all MFI status of all 51 cytokines and seven clinical characteristics, namely sex, race/ethnicity, baseline cirrhosis status, history of hepatic decompensation, and underlying liver disease etiology. For cytokine MFI status, we used a cutoff of above vs. below the median MFI for that individual cytokine and classified MFI as high or low based on that.

To further assess and confirm the robustness of the association of identified biomarker with clinical outcomes, we also conducted re-sampling based tests following previously-described algorithms^32, 33^.

### Data availability

The datasets generated during and/or analyzed during the current study are available from the corresponding author upon reasonable request.

### Disclosures

Vincent Chen, Jacqueline Estevez, An Le, Philip Vutien, Yael Rosenberg-Hasson, and Ellen Chang have no disclosures. Ondrej Podlaha, Biao Li, Stefan Pflanz, Zhaoshi Jiang, Dongliang Ge, and Anuj Gaggar are employees of Gilead Sciences. Mindie Nguyen: research support: Janssen Pharmaceutical, Bristol-Myers Squibb, Gilead Sciences. Consultant and/or an advisory board member: Roche Laboratories, Gilead Sciences, Janssen Pharmaceutical, Intercept Pharmaceutical, Alnynam Pharmaceutical, and Dynavax Laboratories.

## Results

### Overall patient baseline characteristics

Baseline patient characteristics are shown in Table 1. A total of 282 patients were included. The etiology of liver disease was HBV in 112 patients, HCV in 119, and nonviral in 51. HBV patients were primarily Asian (92%) while most HCV and nonviral patients were not (16% and 6%; *p* < 0.0001 for the between-group comparison). Approximately 61% of patients had cirrhosis, and 68% of those with cirrhosis had a history of hepatic decompensation. The overall mean model of end-stage liver disease score was 12.8. HBV patients were less likely to have cirrhosis and had lower model of end-stage liver disease scores.

**Table 1:** Patient baseline characteristics, by liver disease etiology.

Characteristic	HBV (n = 112)	HCV (n = 119)	Non-Viral (n = 51)	*P* value
Age	47.1 ± 12.5	53.5 ± 10.4	58.0 ± 9.8	<**0**.**001**
Male	63.6%	56.9%	55.1%	0.47
Asian	92.4%	17.1%	8.2%	<**0**.**001**
History of alcohol use (n = 148)	40.0%	81.4%	81.4%	<**0**.**001**
History of tobacco use (n = 154)	25.2%	71.3%	53.3%	<**0**.**001**
Cirrhosis	24.6%	85.3%	95.9%	<**0**.**001**
History of decompensation	11.0%	65.5%	89.8%	<**0**.**001**
Ascites	10.2%	52.6%	73.5%	<**0**.**001**
Encephalopathy	4.2%	51.7%	69.4%	<**0**.**001**
Gastrointestinal bleed	5.1%	22.4%	42.9%	<**0**.**001**
White blood cell count (K/μL)	5.8 ± 4.0	6.0 ± 3.6	6.0 ± 2.7	0.88
Hematocrit (%)	42.3 ± 10.6	36.3 ± 8.0	35.0 ± 7.0	<**0**.**001**
Platelet count (K/μL) (n = 172)	202.9 ± 69.9	120.3 ± 74.2	123.9 ± 71.7	<**0**.**001**
International normalized ratio	1.1 ± 0.2	1.4 ± 0.8	1.4 ± 0.4	<**0**.**001**
Creatinine (mg/dL)	0.9 ± 0.2	1.2 ± 1.2	1.1 ± 0.9	**0**.**018**
Total bilirubin (mg/dL)	0.7 (0.5–0.9)	1 (0.6–2.3)	1.5 (0.7–2.8)	**0**.**016**
Aspartate transaminase (U/L)	29 (21–38)	56 (35–100)	38 (29–64)	<**0**.**001**
Alanine transaminase (U/L)	38 (29–50)	51 (32–79)	35 (25–47)	**0**.**013**
Albumin (g/dL)	4.1 (3.8–4.3)	3.3 (2.7–3.8)	3.0 (2.6–3.7)	0.083
Child-Pugh-Turcotte class				
A	88.6%	36.8%	20.4%	<**0**.**001**
B	11.4%	36.0%	49.0%	
C	0%	27.2%	30.6%	
Model of end-stage liver disease	8.3 ± 2.5	13.7 ± 8.7	13.4 ± 6.9	<**0**.**001**

HBV, hepatitis B virus. HCV, hepatitis C virus.

### Overall cumulative HCC incidence

Median follow-up was 65.3 months (IQR 21.5–88.5 months) with a total follow-up period of 1,382 person-years. During this period, there were a total of 25 cases of HCC, 8 in patients with HBV, 13 in patients with HCV, and 4 in patients with nonviral disease. The overall HCC incidence was 18.8 per 1,000 person-years. For HBV, it was 14.6 per 1,000 person-years; for HCV, 23.8 per 1,000 person-years; and for nonviral, 14.8 per 1,000 person-years. For patients with cirrhosis, the incidence was 25.6 per 1000 person-years; and for patients without cirrhosis, 0.6 per 1000 person-years.

### Cytokine profiles and risk for development of HCC

**Figure 1:**
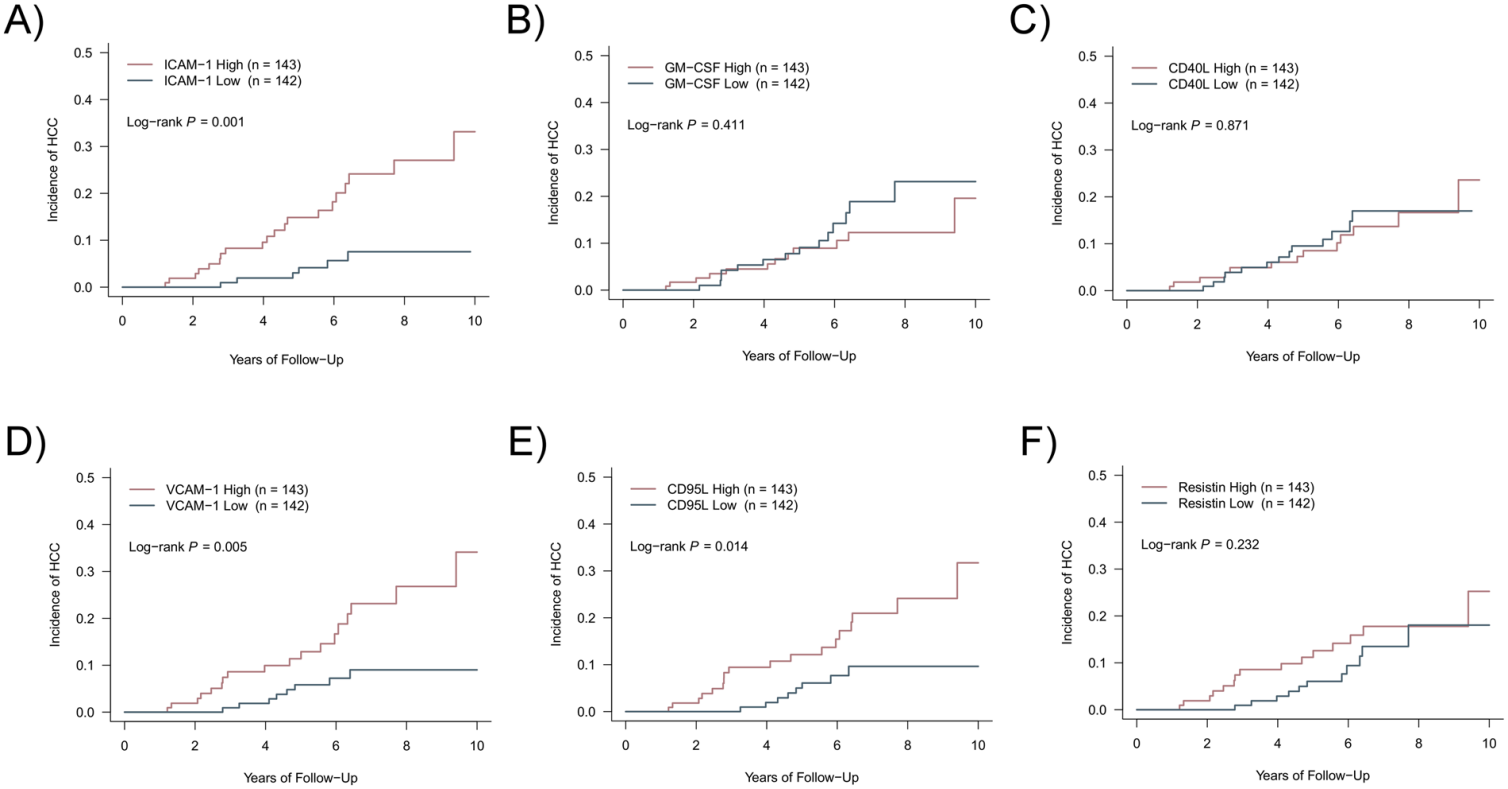
Results of Lasso-based regression to determine parameters associated with hepatocellular carcinoma incidence. (**A**–**F**) Kaplan-Meier curve of hepatocellular incidence based on baseline serum MFI of (A) soluble intercellular adhesion molecule 1, (**B**) granulocyte-macrophage colony stimulating factor, (**C**) CD40 ligand, (**D**) soluble vascular adhesion molecule 1, (**E**) CD95 ligand, and (**F**) resistin. In all panels, “high” denotes MFI above the median for that individual cytokine while “low” denotes MFI below the median.

On Lasso analysis, the parameter most highly correlated with HCC development was history of hepatic decompensation, followed by sICAM-1 MFI, presence of cirrhosis, granulocyte-macrophage colony stimulating factor MFI, and soluble CD40 ligand MFI. On Kaplan-Meier analysis of sICAM-1 and granulocyte-macrophage colony stimulating factor MFI, high sICAM-1 MFI is significantly associated with increased HCC incidence (*p* = 0.001; Fig. 1A) but granulocyte-macrophage colony stimulating factor and CD40 ligand MFI were not (*p* = 0.411 and 0.871, respectively; Fig. 1B and C). Soluble vascular cell adhesion molecule-1, soluble CD95 ligand, and resistin MFI were correlated with sICAM-1 MFI (r = 0.63, 0.53, and 0.51, respectively) and the first two are associated with HCC incidence (*p* = 0.005, 0.014, and 0.232, respectively; Fig. 1D–F). However, sICAM-1 entered the Lasso regression model first, which indicates that it is more strongly associated with HCC incidence than these other three and that the other three do not provide additional predictive information beyond what sICAM-1 provides.

We conducted re-sampling based tests to confirm the robustness of the association of ICAM1 with HCC patient survival rate. Following previously-published algorithms^32, 33^, we randomly selected half of the patient cohort (n = 138) while retaining the proportion of death event as the same as that of the whole cohort. We performed 100,000 times random selection and identified 17 sampling sets that showed less than 50% of overlap among them. For each of these 17 data sets, we ran the same log-rank test as on the whole data set. A total of 14 out of 17 (82%) tests showed a P-value < 0.05. Such a high replication rate from randomly generated data sets confirmed that the expression level of ICAM1 is strongly related to HCC survival probability.

**Figure 2:**
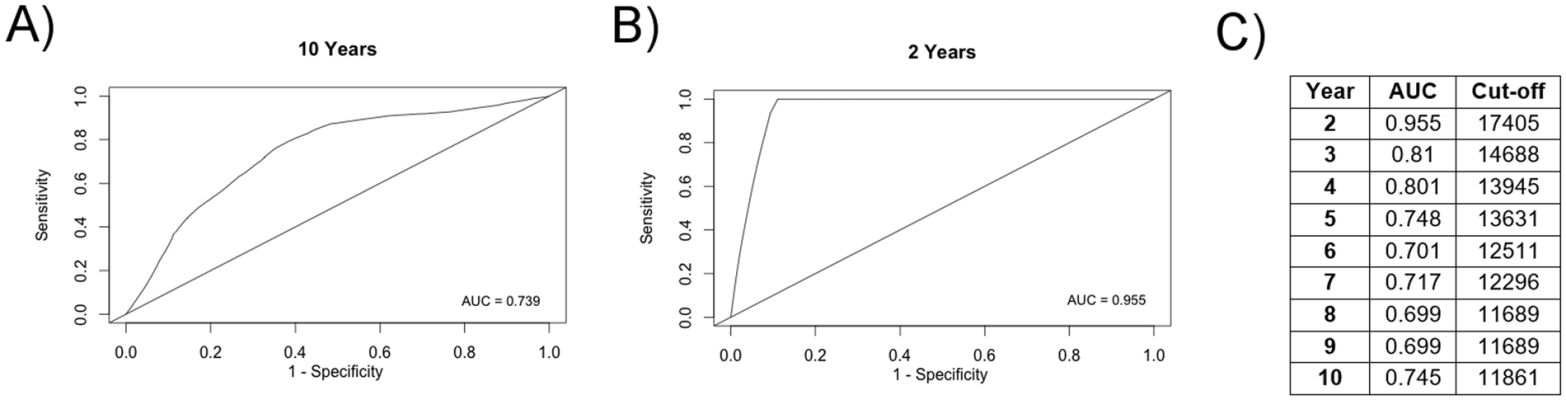
Receiver operating characteristic (ROC) analysis for soluble intercellular adhesion molecule 1 (sICAM-1) and hepatocellular carcinoma (HCC) incidence. (**A** and **B**) ROC curves for association between sICAM-1 and HCC incidence at (A) 10 years with sICAM-1 cutoff of 11,861 mean fluorescence intensity (MFI) and (**B**) 2 years with sICAM-1 cutoff of 17,404 MFI. (**C**) Area under the ROC curve and sICAM-1 cutoff based on duration of follow-up.

**Figure 3:**
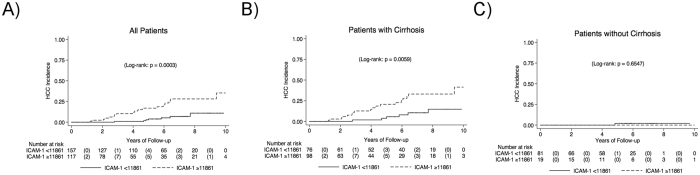
Hepatocellular carcinoma incidence based on soluble intercellular adhesion molecule status. (**A**) Overall cohort. (**B**) Patients with cirrhosis. (**C**) Patients without cirrhosis.

The above HCC incidence analysis used a sICAM-1 cutoff of above vs. below the median (approximately 11,000 MFI). On ROC analysis, sICAM-1 MFI that was most predictive of HCC development within 10 years was at a cutoff of 11,861 MFI with an AUROC of 0.739 (Fig. 2A). At two years, the cutoff was 17,404 MFI with AUROC 0.955 (Fig. 2B), though there were only two events in this time period. Cutoffs for other time points are shown in Fig. 2C. The cutoff appears to decrease over greater length of follow-up. At the cutoff of 11,861 MFI, for the overall cohort, the HR for sICAM-1 MFI and HCC incidence was 4.42 (*p* = 0.0001; Fig. 3A). For patients with cirrhosis, the HR was 3.33 (*p* = 0.011; Fig. 3B) and for patients without cirrhosis there was no association between sICAM-1 and HCC incidence (*p* = 0.69; Fig. 3C); of note the total number of patients without cirrhosis who developed HCC was very low as detailed above. These results indicate that in the overall cohort and in patients with cirrhosis, sICAM-1 MFI is associated with increased HCC incidence. This association persisted with both Asian and non-Asian patients, and with the cutoffs of above vs. below the median and of 11,861 MFI (Supp. Figure 1), in the overall cohort. Supp. Table 1 shows HR for various specified subgroups among patients with baseline cirrhosis. Overall, the associations either were significant or trended toward significance. Several subgroups were not examined, such as patients with nonviral disease, due to low numbers of incident cases of HCC in this group.

### Association of elevated sICAM-1 MFI with patient baseline clinical characteristics

Because of the very low number of new HCC diagnoses among the patients without baseline cirrhosis, we focused for the remainder of our analysis on the patients with baseline cirrhosis. Having identified an association between sICAM-1 MFI and HCC incidence, we next sought to characterize the baseline clinical characteristics of these patients, stratified by sICAM-1 status, among patients with cirrhosis (Table 2). Patients with high sICAM-1 MFI had similar age but were more often male and non-Asian. They also had a greater prevalence of prior hepatic decompensation, higher transaminases, and more advanced liver disease based on platelet count, international normalized ratio, albumin, Child-Pugh score, and model of end-stage liver disease score.

**Table 2:** Baseline characteristics of patients with cirrhosis at entry, by soluble intercellular adhesion molecule-1 status.

Characteristic	sICAM-1 high (n = 98)	sICAM-1 low (n = 75)	*P* value
Age	55.4 ± 9.01	55.0 ± 10.2	0.78
Male	69.4%	53.3%	**0**.**031**
Asian	20.4%	34.7%	**0**.**035**
History of alcohol use	79.8%	68.1%	0.11
History of tobacco use	66.7%	51.4%	0.055
Etiology			
Hepatitis B virus	7.1%	29.3%	<**0**.**001**
Hepatitis C virus	69.4%	37.3%	<**0**.**001**
Non-viral	22.5%	33.3%	0.11
History of decompensation			
Ascites	71.4%	50.7%	**0**.**005**
Encephalopathy	70.4%	40.0%	<**0**.**001**
Gastrointestinal bleed	33.7%	25.3%	0.24
White blood cell count (K/μL)	5.9 ± 3.7	6.5 ± 5.2	0.32
Hematocrit (%)	34.7 ± 7.8	39.8 ± 13.9	**0**.**0023**
Platelet count (K/μL)	102.0 ± 64.0	143.8 ± 79.1	<**0**.**001**
International normalized ratio	1.6 ± 0.8	1.2 ± 0.3	<**0**.**001**
Creatinine (mg/dL)	1.3 ± 0.9	1.3 ± 1.1	0.72
Total bilirubin (mg/dL)	1.5 (0.7–2.8)	1.0 (0.5–2.0)	**0**.**012**
Aspartate transaminase (U/L)	68 (38–111)	34 (28–57)	<**0**.**001**
Alanine transaminase (U/L)	51 (32–88)	34 (24–53)	<**0**.**001**
Albumin (g/dL)	3.1 (2.5–3.6)	3.6 (2.9–3.8)	<**0**.**001**
Child-Pugh-Turcotte class			
A	21.4%	44.0%	<**0**.**001**
B	42.9%	44.0%	
C	35.7%	12.0%	
Model of end-stage liver disease	15.1 ± 9.1	11.4 ± 5.5	**0**.**0022**

sICAM-1, intercellular adhesion molecule-1.

### Elevated sICAM-1 MFI: univariate and multivariate analysis

Univariate analysis of parameters associated with HCC incidence was shown in Table 3 for patients with cirrhosis and Supp. Table 2 for all patients. Among the overall cohort, factors associated with increased HCC incidence were advancing age, presence of cirrhosis, high Child-Pugh score, low platelet count, low albumin, successful treatment with antiviral medications, and high sICAM-1. Most of these parameters are associated with cirrhosis, and among patients with cirrhosis the only factors associated with increased HCC incidence were age, treatment with antiviral agents, and sICAM-1. We constructed a multivariate model that included these three variables, and on multivariate analysis, sICAM-1 remained significantly associated with increased HCC incidence (HR 2.75; *p* = 0.041). Of note, many of the variables that were associated with higher sICAM-1 MFI, as detailed in the previous section, were not themselves associated with increased HCC incidence in patients with cirrhosis.

**Table 3:** Univariate and multivariate analysis: factors associated with hepatocellular carcinoma incidence in patients with cirrhosis.

Characteristic	Unadjusted HR (95% CI)	*P* value	Adjusted HR (95% CI)	*P* value
Age	1.06 (1.01–1.11)	0.010	**1.05 (1.01–1.10)**	**0.022**
Male	1.44 (0.61–3.36)	0.405		
Asian	1.79 (0.80–4.01)	0.155		
Etiology				
Non-viral	Referent	Referent		
Viral	1.96 (0.67–5.75)	0.218		
Child-Pugh score	1.12 (0.95–1.33)	0.187		
Model of end-stage liver disease score	1.01 (0.96–1.07)	0.640		
Alanine transaminase (U/L)	1.01 (0.99–1.01)	0.118		
Bilirubin (mg/dL)	0.97 (0.87–1.07)	0.520		
International normalized ratio	1.10 (0.43–2.82)	0.849		
Creatinine (mg/dL)	1.10 (0.84–1.44)	0.474		
Albumin (g/dL)	0.74 (0.47–1.16)	0.186		
Platelet count (K/µL)	1.00 (0.99–1.01)	0.910		
sICAM-1 MFI (cutoff 11861)	3.33 (1.32–8.40)	**0.011**	**2.75 (1.04–7.26)**	**0.041**
**Antivirals:**				
Nonviral = reference	Referent	Referent		
SVR (HCV) or on antivirals (HBV)	1.04 (0.28–3.88)	0.953		
No SVR or no treatment	2.71 (0.90–8.18)	0.077		
**Antivirals:**				
SVR (HCV) or on antivirals (HBV) or non-viral	Referent	Referent	Referent	Referent
No SVR or no treatment	2.65 (1.16–6.07)	**0.021**	1.96 (0.81–4.70)	0.133

HBV, hepatitis B virus; HCV, hepatitis C virus; MFI, mean fluorescent intensity; sICAM-1, soluble intercellular adhesion molecule-1; SVR, sustained virologic response.

## Discussion

In summary, our comprehensive profiling of 51 cytokines in 282 patients with various chronic liver diseases who were free of HCC at baseline identified sICAM-1 to highly associate with development of future HCC (AUROC 0.74 at year 10 and 0.955 at year 2). These findings held across diverse etiologies of liver disease and in patients with and without cirrhosis, and were independent of established clinical risk factors. To our knowledge, this study is the largest and best-characterized prospective cohort study investigating the cytokine profiles of HCC-free patients with chronic liver disease with respect to hepatic complications including liver cancer development. In addition, it investigated multiple different cytokines in an unbiased way.

Biomarkers to assess survival after HCC development have been comparatively well-established. A seminal paper from 2008 examined gene expression in tumor and peritumor liver tissue in patients treated with partial hepatectomy, and found that peritumor gene signatures were associated with disease recurrence^34^. This research has been extended to biomarkers in serum, which is more easily accessible, especially in patients with advanced-stage HCC who would generally not undergo surgery or liver biopsy. For example, higher serum MFI of macrophage migratory inhibitory factor^35^ and IL-10^36^ is associated with greater likelihood of recurrence in patients treated with partial hepatic resection. A recent analysis of the EVOLVE-1 trial investigating everolimus in HCC patients with tumor progression on sorafenib found that the angiogenesis markers vascular endothelial growth factor and soluble vascular endothelial growth factor receptor 1 were associated with decreased survival^21^. A molecular classification of HCC based on tissue specimens is emerging based on genomic analysis of resected tumor specimens, with three molecularly-defined subclasses of HCC with implications for patient survival^37^. Applying this research to circulating tumor cells raises the possibility of defining molecular subclasses in advanced disease, rather than only early-stage disease. This type of research has important implications in assessing prognosis, identifying biomarkers that might be used to stratify patients in future clinical trials, and discovering potential therapeutic targets for drug development.

In contrast, the field of serum biomarkers to predict clinical outcomes in liver disease remains underdeveloped, though it too has important implications in guiding surveillance frequency: higher-risk patients may warrant more intensive surveillance, while lower-risk patients might safely be surveyed less frequently or with less costly modalities which could decrease healthcare expenditures. The largest study published on serum biomarkers to predict HCC risk is the European Prospective Investigation into Cancer and Nutrition, which prospectively followed over 500,000 healthy individuals and investigated risk factors for liver cancer^6^. This study identified C-reactive protein, IL-6, C-peptide, and non-high weight adiponectin as risk factors for liver cancer. In contrast to ours, this study focused on a healthy population, with a low prevalence of diabetes (under 4%) and no known viral hepatitis or cirrhosis. The most likely risk factor for HCC in this study was non-alcoholic fatty liver disease and HCC incidence was extremely low ( <0.01% per person-year). Other studies investigating biomarkers to predict liver-related outcomes have generally been smaller and limited to a single etiology of disease. For example, IL-6 has been associated with increased HCC risk in chronic hepatitis B^38^, and adiponectin and IL-6 in chronic hepatitis C^39^. There are multiple well-established models for predicting HCC risk in chronic hepatitis B as well as chronic hepatitis C albeit to a lesser extent for the latter^14–16^, but these models have mostly been developed in restricted geographic regions and are not universally applicable across regions or ethnicities. In addition, risk calculators for nonviral liver disease are much less developed. Therefore, further research to enhance current risk prediction for HCC in diverse patient population is needed and novel biomarker with high predictive accuracy and precision can improve the current diagnostic strategies for HCC as well as therapeutic and surveillance strategies for high-risk patients.

In regards to sICAM-1, there is biologic rationale underlying its association with clinical outcomes such as HCC. sICAM-1 is correlated with more advanced tumor stage in humans though mechanisms for this finding are not well-established^28^. Potential explanations include that ICAM-1 expressed by tumor cells may help cancer cells adhere to leukocytes and enter the blood stream; sICAM-1 inhibits interactions between tumor-specific T cells and cancer cells; and sICAM-1 shed by tumor cells promotes angiogenesis^23, 25–27^. Surface ICAM-1 helps regulate leukocyte extravasation, It may also inhibit the host immune response to malignant cells^23^. However, the mechanism behind the association between sICAM-1 MFI in cancer-free patients and HCC incidence is even more speculative and may relate to sICAM-1 production by pre-malignant HCC stem cells^24^.

Our study has several strengths. First, it is relatively large and includes patients of diverse racial/ethnic backgrounds, with different etiologies of disease, and with different severities of underlying liver disease. In addition, the method detailed here is practical since it is based on readily-available serum rather than tissue. The selection of cytokines and other serum markers was unbiased in that it was not selected for specific proteins known to be involved in the pathogenesis of liver disease. There are also several limitations of this study. It included only cytokines and other proteins related to immune function, while there are a number of other pathways known to be implicated in HCC development, including oxidative stress and numerous other modulators of metabolism^40, 41^. Finally, this cohort was too small to investigate how cytokines affected HCC risk in patients without cirrhosis, who had a low overall incidence of HCC, so future larger studies will be required to assess biomarkers as risk factors for HCC in this lower-risk population.

In conclusion, this is to our knowledge the largest and most diverse prospective study of patients with chronic liver disease examining associations between longitudinal clinical outcomes and the baseline levels of serum cytokines and other biomarkers. We have identified sICAM-1 as a biomarker associated with HCC incidence with chronic liver disease. Use of these biomarkers could potentially guide decisions surrounding HCC surveillance frequency and therapeutic decision for antiviral therapies in higher-risk patients with viral hepatitis. Future studies will be required to validate these findings, extend them to patients without liver cirrhosis, and create a predictive model for HCC that includes sICAM-1.

## Electronic supplementary material

Supplemental Information
