# Supplementary material for: Soluble intercellular adhesion molecule-1 is associated with hepatocellular carcinoma risk: multiplex analysis of serum markers
Source: Sci Rep. 2017 Sep 11;7:11169. doi: 10.1038/s41598-017-10498-5 (PMC5593940; doi:10.1038/s41598-017-10498-5)

Title: Soluble intercellular adhesion molecule-1 is associated with hepatocellular carcinoma risk: multiplex analysis of serum markers

Authors: Vincent L. Chen,[1,2] An K. Le,[3] Ondrej Podlaha,[4] Jacqueline Estevez,[3,5] Biao Li,[4] Philip Vutien,[6] Ellen T. Chang,[7] Yael Rosenberg-Hasson,[8] Stefan Pflanz,[4] Zhaoshi Jiang,[4] Dongliang Ge,[4] Anuj Gaggar,[4] Mindie H. Nguyen[3]

Supplementary Table 1: Association between high serum soluble intercellular adhesion molecule-1 status (above vs. below 11,861 mean fluorescence intensity units) with hepatocellular carcinoma incidence among subgroups of patients with baseline cirrhosis

**Subgroup**	**HR (95% CI)**	***P* value**
**Viral**	2.18 (0.83 – 5.67)	0.11
**ALT < ULN**	3.99 (0.36 – 44.32)	0.26
**ALT > ULN**	2.86 (1.05 – 7.83)	**0.041**
**Asian**	3.63 (0.96 – 13.68)	0.057
**Non-Asian**	3.15 (0.86 – 11.53)	0.084
**Age > 60**	11.04 (1.40 – 86.93)	**0.023**
**Age < 60**	1.82 (0.59 – 5.58)	0.30

ALT, alanine transaminase; ULN, upper limit of normal

Supplementary Table 2: Univariate analysis: factors associated with hepatocellular carcinoma incidence in all patients

Characteristic	Unadjusted HR (95% CI)	*P* value
**Age**	1.06 (1.02 – 1.10)	**0.003**
**Male**	1.53 (0.66 – 3.55)	0.320
**Asian**	0.92 (0.42 – 2.03)	0.845
**Etiology**		
Non-viral	Referent	Referent
Viral	1.21 (0.41 – 3.54)	0.727
**Cirrhosis**	12.78 (1.72 – 94.65)	**0.013**
**Child-Pugh score**	1.25 (1.08 – 1.45)	**0.003**
**Model of end-stage liver disease score**	1.04 (0.99 – 1.09)	0.163
**Alanine transaminase (U/L)**	1.00 (0.99 – 1.01)	0.331
**Bilirubin (mg/dL)**	0.98 (0.90 – 1.07)	0.641
**International normalized ratio**	1.68 (0.76 – 3.70)	0.197
**Creatinine (mg/dL)**	1.18 (0.91 – 1.52)	0.206
**Albumin (g/dL)**	0.57 (0.38 – 0.86)	**0.007**
**Platelet count (K/µL)**	0.994 (0.98 – 0.999)	**0.048**
**sICAM-1 MFI (cutoff 11,861)**	4.42 (1.84 –10.61)	**0.001**
**Antivirals:**		
Nonviral = reference	Referent	Referent
SVR (HCV) or on antivirals (HBV)	0.58 (0.15 – 2.16)	0.416
No SVR or no treatment	1.82 (0.61 –5.46)	0.285
**Antivirals:**		
SVR (HCV) or on antivirals (HBV) or non-viral	Referent	Referent
No SVR (HCV) or no antivirals (HBV)	2.52 (1.11 – 5.71)	**0.027**

HBV, hepatitis B virus; HCV, hepatitis C virus; MFI, mean fluorescent intensity; sICAM-1, soluble intercellular adhesion molecule-1; SVR, sustained virologic response

Supplementary Figure 1: Hepatocellular carcinoma incidence based on soluble intercellular adhesion molecule-1 (sICAM-1) status, stratified by race. (A-B) Incidence in Asian (A) or non-Asian (B) patients, based on sICAM-1 mean frequency intensity above vs. below the median. (C-D) Incidence in Asian (C) or non-Asian (D) patients, based on sICAM-1 mean frequency intensity above vs. below 11,861.

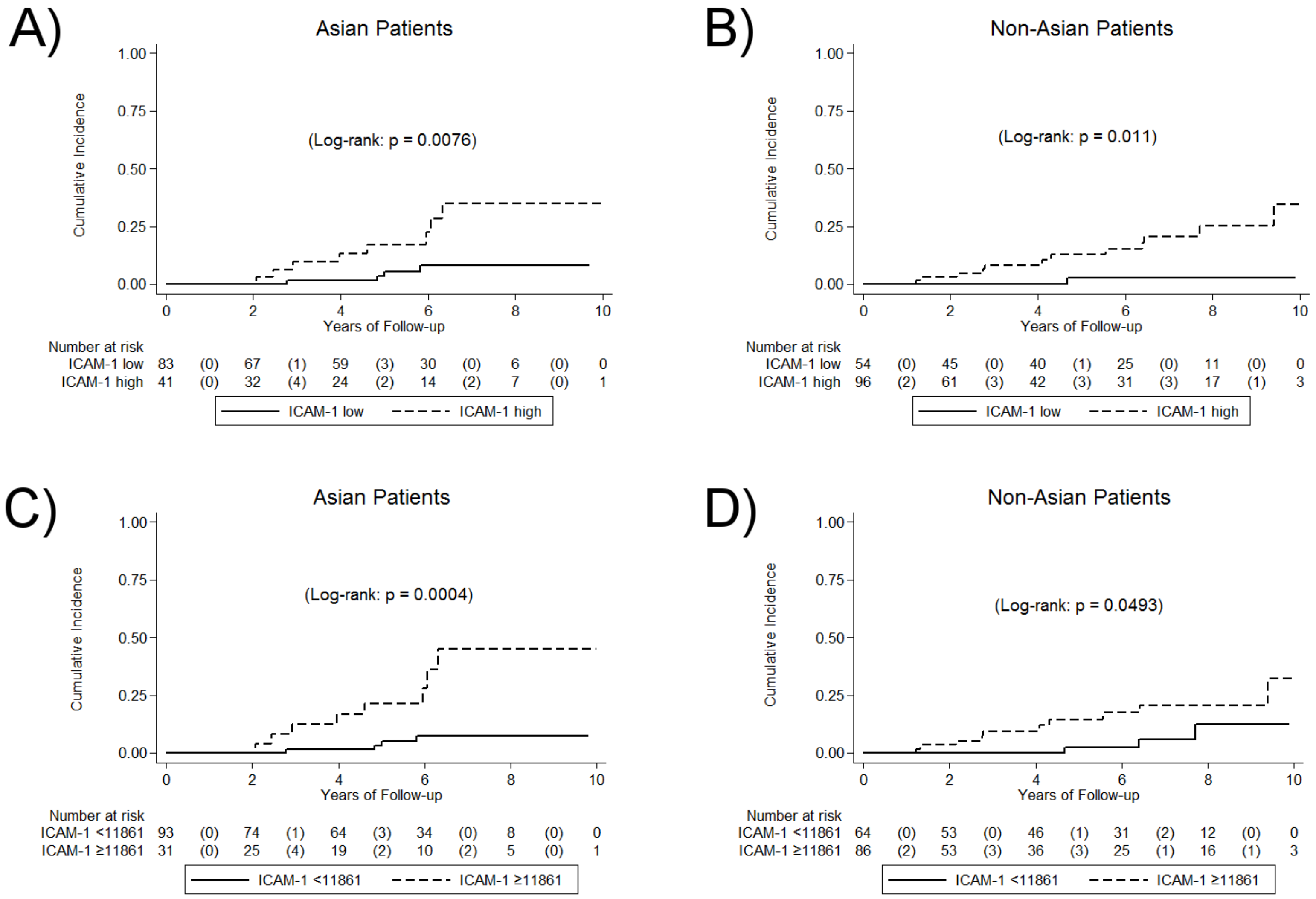

Supplement: Supplementary file 1 — Supplemental Information [file 41598_2017_10498_MOESM1_ESM.pdf]
